# Transcriptomic Analysis of Cold-Induced Temporary Cysts in Marine Dinoflagellate *Prorocentrum cordatum*

**DOI:** 10.3390/ijms26125432

**Published:** 2025-06-06

**Authors:** Mariia Berdieva, Pavel Safonov, Olga Palii, Mikhail Prilutsky, Olga Matantseva, Sergei Skarlato

**Affiliations:** 1 Laboratory of Cytology of Unicellular Organisms, Institute of Cytology of the Russian Academy of Sciences, St. Petersburg 194064, Russia; safonov.incras@gmail.com (P.S.); volchanka1996@gmail.com (O.P.); mhl981622@gmail.com (M.P.); sergei.skarlato@mail.ru (S.S.); 2 Central Collection of Algal Cultures, Faculty of Biology, University of Duisburg-Essen, 45141 Essen, Germany; olga.matantseva@uni-due.de

**Keywords:** cold stress, cyst, dinoflagellate, MEI2, transcriptome

## Abstract

Dinoflagellates are unicellular organisms that are crucial components of aquatic ecosystems, known as important primary producers and causes of harmful blooms. They have complex life cycles, including immotile stages, which contribute to their distribution and survival in unfavorable conditions. Temperature changes, primarily cold stress, significantly impact dinoflagellate physiology, influencing metabolic processes, growth rates, and encystment/excystment cycles. This study investigates the transcriptome of temporary cold-induced cysts in the marine planktonic dinoflagellate *Prorocentrum cordatum*. We compared gene expression in cysts subjected to a 7-h cold incubation with those returned to standard cultivation conditions and motile vegetative cells. Our results showed a marked predominance of downregulated genes in cold-induced cysts. Encystment affected signaling pathways, including calcium and protein kinase signaling, as well as RNA and protein metabolism. Upon returning to standard conditions, RNA metabolism was reactivated; upregulation of genes encoding some calcium-binding proteins and kinases was observed. Additionally, we analyzed RNA-binding pentatricopeptide repeat-containing proteins, the genes encoding which changed their expression in *P*. *cordatum* cysts, for similarities to plant MRL1 proteins. Finally, we focused on MEI2-like proteins to confirm their role in non-sexual cyst formation and position them within the diversity of MEI2 homologs in dinoflagellates.

## 1. Introduction

Dinoflagellates exhibit complexity and variability in their life cycle routes, which is one of the reasons for their ubiquitous distribution and adaptive plasticity. These microalgae can alternate between vegetative and sexual reproduction and can enter various immotile stages. These life cycle transitions determine the formation and development of dinoflagellate blooms (harmful algal blooms, HABs)—hazardous phenomena that occur in the coastal waters worldwide and pose a threat for marine communities, aquacultures, and human health. The initiation of a bloom can occur either through the intensive division of vegetative cells or due to the simultaneous release of cells from cysts accumulating in bottom sediments. These cysts can originate from either the sexual or vegetative stage [1,2,3]. During subsequent growth and maintenance of the bloom, not only multiple asexual divisions but also sexual reproduction and the short-term encystment/excystment dynamics can promote proliferation [2,3,4]. In some cases, bloom termination is associated with the encystment of dinoflagellates [5]. The information about the transitions in the dinoflagellate life cycles, along with understanding the exogenous and endogenous factors that regulate them, enables modeling of bloom development under specific conditions. However, the molecular mechanisms underlying such regulation remain poorly understood, yet they are crucial for comprehending patterns of life-history stage alternation and particularities of response of dinoflagellate cells to environmental changes. Additionally, this information could be used in developing molecular tools for the rapid analysis of processes observed in natural dinoflagellate populations. The search for molecular bloom markers is a promising area of research, with genes involved in life cycle regulation considered as potential candidates [6,7,8].

Dinoflagellate cysts are classified into two primary types. The first one, resting cysts, is characterized by a significant period of dormancy (long-term), which can reach several months or even years, a wall of 1–3 layers (thick-walled) containing specific carbohydrate-based biopolymers dinosporins, and drastic alterations in internal cell organization [9,10]. The second type is cysts, which do not undergo prolonged dormancy (short-term, or temporary), they stay immotile for a few hours or days, although sometimes for months [9]. These cysts have a thin wall, typically formed by a pellicle—complex layer of still unclear nature [9,11]; in the absence of a pellicle, some armored species form thecate cysts that retain cellulose thecal plates as a “wall” [4,12]. Dinoflagellates employ both sex-associated and sex-independent encystment strategies [9]. The former occurs during sexual reproduction as a possible dormancy period following zygote formation. Conversely, vegetative cells can also form cysts as a survival strategy in response to various stressors, including temperature changes, oxidative stress, low light conditions, and mechanical impacts [12,13,14,15,16,17,18]. Cold stress is considered a significant factor, influencing dinoflagellate growth and life cycle strategies, including the encystment/excystment alternation. A decrease in temperature can impact bloom development by delaying excystment and promoting encystment [2,19].

In this study, we present the results of the first transcriptome analysis of cold-induced temporary cysts in marine planktonic, bloom-forming, armoured dinoflagellate *Prorocentrum cordatum*. This eurythermal species has a pan-global distribution [20], and understanding *P*. *cordatum* cell response to low temperatures is essential for elucidating its survival strategies and ecological impact. We compared gene expression in cysts after a 7-h incubation in cold with that in cysts returned to standard cultivation conditions and with motile vegetative cells. The main patterns of expression changes were characterized.

Additionally, we focused on MEI2-like proteins encoded by differentially expressed genes. MEI2 was described as a regulator of meiosis initiation in the fission yeast *Schizosaccharomyces pombe* [21]. In plants, genes encoding MEI2-like proteins are active in both vegetative and generative tissues [22]. Dinoflagellates are notable for possessing a large number of MEI2 homologs [3,23]. While there is some evidence that MEI2-coding genes exhibit expression changes during encystment in dinoflagellates [13,24], it remained unclear whether their function is related to the sexual process. Our data demonstrate that MEI2-like proteins are indeed involved in the non-sexual formation of temporary cysts in dinoflagellates. We also analyzed their position within the diversity of MEI2 homologs in dinoflagellates.

## 2. Results

### 2.1. Induction of Encystment

**Figure 1:**
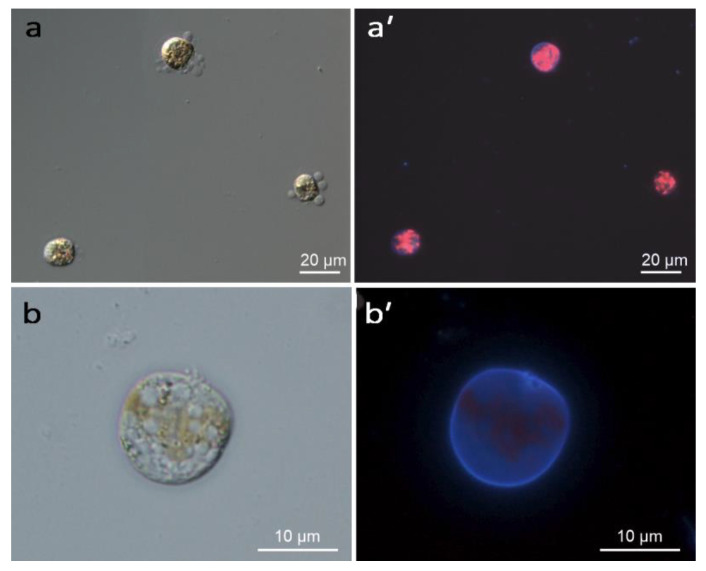
Light microscopy of cold-induced cysts of *Prorocentrum cordatum* at 40× (**a**) and 100× (**b**) magnification. Cellulose thecal plates are stained blue with Calcofluor White M2R (**a′**,**b′**). The red signal represents chloroplast autofluorescence.

Incubation in cold temperature induced encystment of *Prorocentrum cordatum* cells. The cold-induced cysts were stained with Calcofluor White M2R (Figure 1). The percentage of stained cells was 87.9% ± 0.04% (mean ± standard error, SE) in samples after incubation at 2 °C for 7 h (“Cold 7 h”), compared to 2.7% ± 0.004% (mean ± SE) in the control samples. The mortality rate of cold-treated cells was 21.1% ± 0.002% (mean ± SE).

### 2.2. RNA Sequencing Results

RNA sequencing was performed on nine RNA samples—three from each treatment group. On average, 214,652,629 raw reads, with adapters removed, were obtained per sample (Table S1). The mean Q30 value indicating the base calling accuracy score was ≥92.26%. After removing low quality reads, an average of 206,378,131 clean reads per sample (~20.8 Gb) were obtained and further processed for genome-guided transcriptome assembly (Table S1). The clean reads were mapped to the *P*. *cordatum* CCMP1329 reference genome presented by Dougan with co-authors [25]. In the analysis of differential gene expression, a total of 49,566 genes were included (Table S2).

### 2.3. Differential Gene Expression Analysis

**Figure 2:**
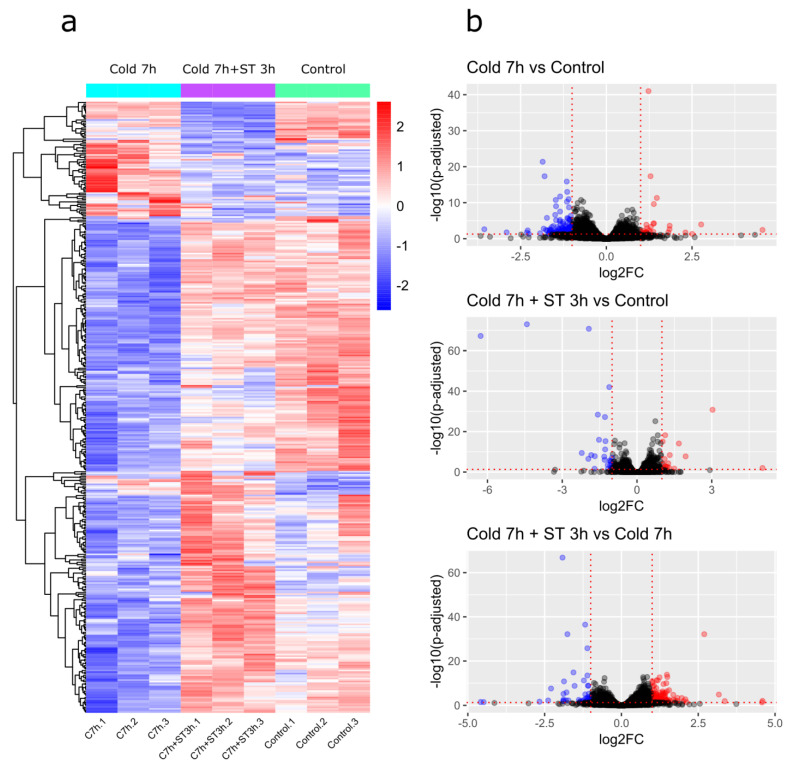
(**a**) Hierarchical clustering heatmap of DEGs in Cold 7 h, Cold 7 h + ST 3 h, and Control (with 3 replicates for each condition). A total of 384 genes with padj < 0.05 and |log_2_ fold change| > 1 were analyzed. The color scale represents row z-scores of log_2_-transformed TPM + 1 values. The distance matrix was calculated using Pearson correlation, and clusters were generated using the complete linkage (furthest neighbor) method. (**b**) Volcano plots depicting differential gene expression between cells subjected to cooling for 7 h and cells from the control group (Cold 7 h vs. Control), between cells cooled for 7 h and then incubated under standard cultivation conditions for 3 h, and cells from the control group (Cold 7 h + ST 3 h vs. Control), and between the two experimental groups (Cold 7 h + ST 3 h vs. Cold 7 h). Genes with padj < 0.05 and |log_2_ fold change| > 1 are marked in color: blue dots indicate downregulated genes, while red dots indicate upregulated genes. Black dots indicate genes whose expression did not change significantly. The dotted lines mark the threshold values.

To analyze patterns of gene expression in the acquired transcriptomes, we have conducted a hierarchical clustering of the 384 revealed differentially expressed genes (DEGs) (Figure 2a). These were all unique genes that significantly changed expression in analysis (|log_2_ fold change| > 1 and padj < 0.05). The results confirmed pronounced differences in transcriptomic profiles between Cold 7 h and Control and between Cold 7 h and Cold 7 h + ST 3 h groups. The profiles of Cold 7 h + ST 3 h and Control groups were more similar, however, certain sub-clusters of genes were also differentially expressed in these two groups.

We performed pairwise comparisons of gene expression between each experimental group and the control group (Cold 7 h vs. Control and Cold 7 h + ST 3 h vs. Control), as well as between the experimental groups themselves (Cold 7 h + ST 3 h vs. Cold 7 h). A total of 182 genes significantly changed their expression in cold-induced cysts compared to non-treated cells, of which 27 were upregulated and 155 were downregulated (Cold 7 h vs. Control, Figure 2b; Table S3). When we compared the expression of genes in cysts returned to standard cultivation conditions with non-treated cells, we identified 45 DEGs—18 upregulated and 27 downregulated (Cold 7 h + ST 3 h vs. Control, Figure 2b; Table S4). In the comparison of expression profiles of cysts returned to standard conditions against those after 7-h incubation, 241 DEGs were detected, of which 195 were upregulated and 46 were downregulated (Cold 7 h + ST 3 h vs. Cold 7 h, Figure 2b; Table S5).

The principal component analysis (PCA) score plot revealed a distance between the control and experimental groups and similarity between samples within them. For the comparison of Cold 7 h vs. Control, PC1 and PC2 accounted for 46% and 19% of the variance, respectively (Figure S1a). In the comparison Cold 7 h + ST 3 h vs. Control, they accounted for 35% and 24% (Figure S1b). Lastly, for the comparison of Cold 7 h + ST 3 h vs. Cold 7 h, PC1 and PC2 explained 54% and 16% of the variance, respectively (Figure S1c).

#### 2.3.1. Cold 7 h vs. Control

Cold-induced cysts demonstrated a substantial predominance of downregulated genes (Table S3). We identified homology or the presence of conserved domains/protein family membership for 93 downregulated DEGs. Among them, several groups of genes were distinguished (Table S3). The **first group** included five genes encoding Ca^2+^-binding EF domain-containing proteins (CAK0885479.1, fold change (FC) = 0.48; CAK0816362.1, FC = 0.40; CAK0876910.1, FC = 0.39; CAK0853012.1, FC = 0.36; CAK0862302.1, FC = 0.45). The **second** and **third group** both contained genes coding for RNA-binding proteins, however, we separated them due to potentially different functional activity. The **second group** included 32 DEGs encoding proteins containing RNA-binding pentatricopeptide repeat(s) (PPR), such as CAK0865768.1 (FC = 0.50) and CAK0873466.1 (FC = 0.34). The **third group** consisted of five DEGs coding for MEI2-like proteins, which contain RNA recognition motif (RRM domain), namely CAK0884715.1 (FC = 0.48), CAK0887363.1 (FC = 0.45), CAK0909770.1 (FC = 0.39), CAK0844294.1 (FC = 0.34), and CAK0818609.1 (FC = 0.33). The **fourth group**, consisting of three DEGs, included proteins involved in protein metabolism, such as homologs of peptidases S8 (CAK0847251.1, FC = 0.46; CAK0847253.1, FC = 0.40) and M11 (CAK0824606.1, FC = 0.29). The **fifth group** was represented by 10 DEGs identified as putative members of protein kinase family, and more specifically, serine/threonine protein kinases (e.g., CAK0887519.1, FC = 0.48; cAMP-dependent protein kinase homolog CAK0819923.1, FC = 0.47). Besides, we detected downregulated DEGs coding for homolog of phospholipase C-like phosphodiesterase (CAK0893731.1, FC = 0.49), sequences similar to cytochrome b2 (CAK0839236.1, FC = 0.33; CAK0839237.1, FC = 0.32), Na^+^/H^+^ exchanger (CAK0857021.1, FC = 0.43), and 14-3-3 regulatory protein (CAK0790679.1, FC = 0.34).

Among 27 upregulated DEGs (Table S3) we identified homologs of acetyl-CoA C-acetyltransferase (CAK0894971.1, FC = 3.5), transcription and mRNA export factor ENY2 (CAK0895837.1, FC = 5.67), a subunit of the, presumably, AP-4 adaptor complex (CAK0798432.1, FC = 2.46), and chaperone protein DnaJ (CAK0834093.1, FC = 2.25).

Gene Ontology (GO) terms enrichment analysis confirmed that the most enriched GO term in the Cold 7 h vs. Control comparison was related to RNA binding (“Molecular Function” category, GO:0003723, adjusted *p*-value = 8.65 × 10^−7^).

#### 2.3.2. Cold 7 h + ST 3 h vs. Control

Cells returned to standard cultivation conditions demonstrated slightly pronounced differences in gene expression compared to non-treated cells (Table S4). We revealed upregulation of two genes encoding proteins related to photosynthesis, namely light harvesting complex proteins (CAK0896970.1, FC = 3.87; CAK0906433.1, FC = 2.04), and two genes coding for proteins containing an RRM domain and similar to MEI2-like proteins (CAK0836878.1, FC = 2.16; CAK0828848.1, FC = 2.09). Additionally, the expression of genes encoding an SPFH domain-containing protein (CAK0899542.1, FC = 32.84), an NAD(P)H-binding domain-containing protein (CAK0911986.1, FC = 8.18), a PAS domain-containing protein (CAK0908113.1, FC = 2.25), as well as cysteine hydrolase (CAK0851015.1, FC = 2.38) and a homolog of a tellurium resistance protein (CAK0901726.1, FC = 2.11) was increased.

Among DEGs with decreased expression, genes related to various molecular machineries were detected (Table S4). In particular, we identified DEGs encoding different transferase: a homolog of DHHC domain-containing palmitoyltransferase (CAK0823863.1, FC = 0.49), a putative glycosyltransferase (CAK0845334.1, FC = 0.41), and a member of the SET domain-containing methyltransferase family (CAK0816234.1, FC = 0.26). Moreover, another MEI2-like RRM domain-containing protein was detected (CAK0884998.1, FC = 0.49); expression of genes coding for homologs of peptidase M11 (CAK0824606.1, FC = 0.41) and Ran GTPase-activating protein (CAK0804354.1, FC = 0.46) was also downregulated. Notably, we revealed a sequence similar to viral reverse transcriptase that showed a drastic decrease in expression (CAK0813222.1, FC = 0.01).

#### 2.3.3. Cold 7 h + ST 3 h vs. Cold 7 h

When we compared expression patterns in cold-induced cysts and cells returned to standard conditions (Table S5), we revealed numerous genes with enhanced expression, including two genes encoding SPFH domain-containing proteins (CAK0899542.1, FC = 24.13; CAK0869958.1, FC = 10.29), gene, encoding a membrane fatty acid desaturase (CAK0840549.1, FC = 23.84), genes of two MEI2-like RRM domain-containing proteins (CAK0884715.1, FC = 2.48; CAK0818609.1, FC = 2.45), two genes encoding axonemal dynein heavy chain proteins (CAK0888335.1, FC = 2.15; CAK0856340.1, FC = 2.02), and a **group** of four Ca^2+^-binding EF domain-containing proteins (CAK0853012.1, FC = 2.75; CAK0887661.1, FC = 2.6; CAK0790159.1, FC = 2.45; CAK0882003.1, FC = 2.14). Notably, CAK0853012.1 was downregulated in cold-induced cysts (Cold 7 h vs. Control FC = 0.36). We also revealed a large **group** comprising 29 DEGs encoding PPR-containing proteins, of which 14 transcripts were downregulated in cold-induced cysts (Table S5).

Additionally, among the upregulated DEGs, we distinguished a **group** of four genes encoding proteins that contain multiple signal sensor PAS domains (PAS-fold, specifically). The sequences are as follows: CAK0900949.1 (FC = 2.68), CAK0908113.1 (FC = 2.93), CAK0869931.1 (FC = 2.58), and CAK0908112.1 (FC = 2.00). These sequences did not possess confidently recognized associated domains that facilitate their identification. CAK0900949.1 only displayed non-specific hits with the COG5809 “Sporulation sensor histidine kinase E” domain family, although this is primarily associated with bacteria. The blastp analysis also revealed similarities to several histidine kinases.

There were DEGs that exhibited decreased expression in this comparison (Table S5). We were able to predict putative function or, at the very least, identify the presence of conserved domains for 16 of these DEGs. For example, genes encoding another fatty acid desaturase (CAK0875354.1, FC = 0.47) and a cytoplasmic dynein light chain (CAK0865836.1, FC = 0.50) were downregulated. Furthermore, some genes that were suppressed in the comparison Cold 7 h + ST 3 h vs. Control were also detected here. These included genes encoding homologs of glycosyltransferase (CAK0845334.1, FC = 0.47), SET domain-containing methyltransferase (CAK0816234.1, FC = 0.26), and Ran GTPase-activating protein (CAK0804354.1, FC = 0.46). Additionally, we identified a sequence corresponding to Arf GTPase activating protein (CAK0852999.1, FC = 0.48).

GO enrichment analysis confirmed the GO term related to RNA binding (“Molecular Function” category, GO:0003723) as the most enriched also in the comparison Cold 7 h + ST 3 h vs. Cold 7 h (adjusted *p*-value = 0.01).

### 2.4. MEI-like Sequences Analysis

**Figure 3:**
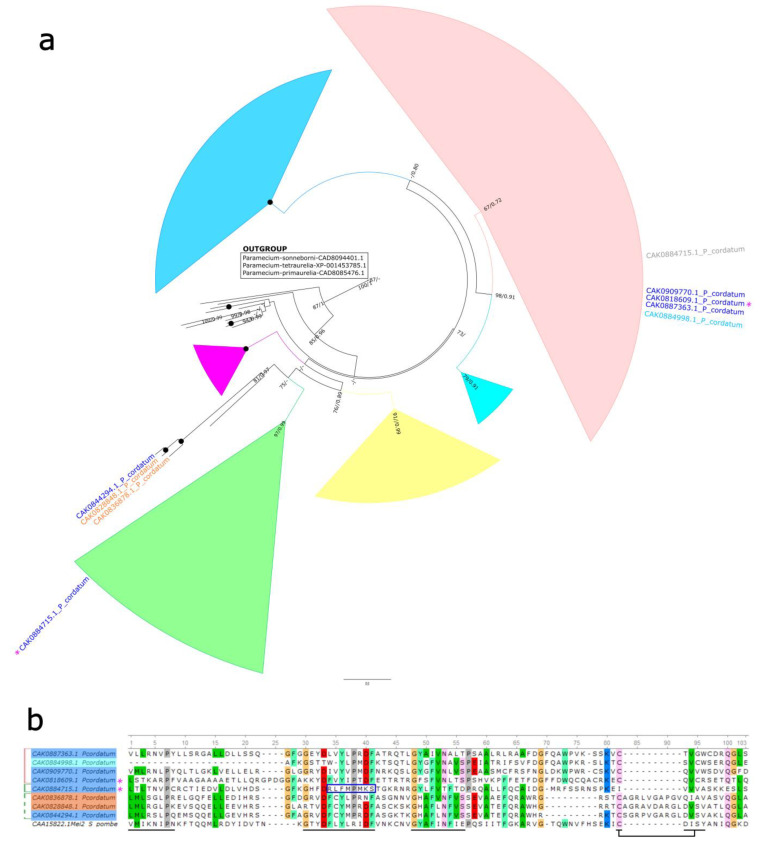
(**a**) Rooted maximum likelihood phylogenetic tree of MEI2 homologs from dinoflagellates, inferred using Q.pfam+R8 evolutionary model. Bootstrap values (based on 10.000 ultrafast replicates) and Bayesian posterior probabilities are indicated at the nodes (not shown when below 60/0.90). Black circles indicate nodes with full support (bootstrap value/posterior probability =100/1.00). MEI2-like sequences from ciliates were used as an outgroup. The reconstruction is based on the dataset from Palii et al. [23], supplemented with eight sequences of *P*. *cordatum* encoded by genes significantly differentially expressed in cold-induced cysts and in cells returned to standard cultivation conditions. Only the names of these sequences and those comprising the outgroup are shown. Clade color coding corresponds to Figure 2 in Palii et al. [23]. *P*. *cordatum* sequences corresponding to DEGs downregulated in the comparison Cold 7 h vs. Control are highlighted in blue, sequences also corresponding to DEGs upregulated in the comparison Cold 7 h + ST 3 h vs. Cold 7 h are labeled with purple asterisks. The sequence CAK0884715.1, highlighted in gray, is duplicated in accordance with the position inferred by Bayesian analysis. Sequences corresponding to DEGs upregulated and downregulated in the comparison Cold 7 h + ST 3 h vs. Control are highlighted in orange and cyan, respectively. (**b**) Fragment of the multiple sequence alignment of the eight MEI2-like sequences from *P*. *cordatum* corresponding to DEGs and the MEI2 sequence from the fission yeast *Schizosaccharomyces pombe*. This fragment corresponds to the RRM3 domain. Key conserved structural and functional sites in the β-strand regions, potentially involved in RNA binding are underlined. A modification in the second site of the sequence CAK0884715.1 is indicated with a blue border. Highlighting and labels follow the same format as in (**a**).

To define the features of MEI2-like sequences detected in our experiment and to specify their position among other dinoflagellate homologs, we performed additional analysis. The phylogeny was inferred using a primary dataset we gathered earlier [23], supplemented with sequences from *P*. *cordatum* identified as significant DEGs. Previously, we defined six putative groups of dinoflagellate MEI2-like proteins [23]. According to the obtained phylogenetic tree, three out of five proteins (CAK0887363.1, CAK0909770.1, and CAK0818609.1) encoded by downregulated DEGs in cold-induced cysts (Cold 7 h vs. Control) clustered in one clade (Figure 3a). The position of the fourth sequence CAK0884715.1 differed in the phylogenies based on maximal likelihood and Bayesian analysis; it was either placed in the same clade as the three aforementioned sequences or in a separate clade of MEI2-like proteins (Figure 3a). Moreover, this sequence exhibited a long branch. The fifth sequence CAK0844294.1 was in the branch basal to this clade (Figure 3a). In the comparison Cold 7 h + ST 3 h vs. Cold 7 h, genes encoding CAK0884715.1 and CAK0818609.1 showed an increase in expression. Three DEGs exhibited significant changes in expression only in comparison Cold 7 h + ST 3 h vs. Control. When we compared the expression patterns between Cold 7 h + ST 3 h and Cold 7 h, the trends for these DEGs were similar, but the changes were not statistically significant. One of these (CAK0884998.1), which was downregulated, clustered with three sequences downregulated in cysts (Figure 3a). Two sequences of upregulated DEGs (CAK0836878.1, CAK0828848.1) branched together with CAK0844294.1 (Figure 3a).

Additionally, we performed multiple sequence alignment of these sequences with the canonical MEI2 sequence of fission yeast *Schizosaccharomyces pombe* (Figure 3b). The alignment focused on the RRM3 (RNA recognition motif 3) domain, as dinoflagellate homologs contain only this RRM domain. Key specific sites corresponding to β-strands, which are expected to interact with RNA, remained largely conserved, with a few exceptions (Figure 3b, underlined). We observed patterns in amino acid composition that are consistent with clustering of sequences. For example, sequences CAK0836878.1, CAK0828848.1, and CAK0844294.1 exhibited changes in the C-terminus of the domain (alignment positions 70–92). In the sequence CAK0884715.1, a modification at the second site was noted (Figure 3b, blue border), which appears to explain the long branch and unstable position in the phylogenetic tree.

## 3. Discussion

Temperature changes are an important external trigger that impacts physiology and ecological dynamics of dinoflagellates. Cold stress induces various responses in these microalgae, including alterations in metabolic processes, changes in membrane fluidity, and shifts in photosynthetic efficiency [13,26,27,28]. Such stressors can affect their growth rates and life cycle progression [28,29]. One of the responses to a decrease in temperature is encystment. *Scrippsiella acuminata* (syn. *S*. *trochoidea*) and *Lingulaulax polyedra* (syn. *Lingulodinium polyedrum, Gonyaulax polyedra*) have been shown to form pellicle cyst in response to cold and darkness [13,14,15]. Grzebyk and Berland [16] observed encystment of *Prorocentrum cordatum* (syn. *P*. *minimum*) within 24 h after transferring cells from 18 °C to 10 °C. The authors described these cysts as pellicular. Matantseva and co-authors [12] investigated the response of *P*. *cordatum* to a short-term cold shock (20 min on ice), but they did not observe a pellicular layer in the resulting temporary cysts and considered them thecate cysts.

We analyzed the transcriptome of *P. cordatum* cold-induced cysts. As expected, the suppression of some molecular processes was observed. Encystment affects various signaling pathways, including calcium and protein kinase signaling, as well as RNA and protein metabolism. When cells were transferred back into standard cultivation conditions, i.e., presumably during preparation for excystment and restoration of normal physiology, RNA metabolism and processing were re-activated, upregulation of calcium binding proteins occurred. The expression of genes encoding signal sensor PAS-domains-containing proteins, which could presumably be histidine kinases, increased. The activity of genes coding for axonemal dynein proteins may reflect preparation for new flagella assembly. Notably, fatty acid desaturase was expected to be highly expressed during cold treatment, since higher proportions of unsaturated fatty acids are assumed for dinoflagellate cells in low-temperature conditions [27]; however, we identified such DEGs in cells returned to standard conditions. Next, we would like to consider several aspects of cold-induced encystment in *P. cordatum* in more detail.

A significant proportion of DEGs encoded putative components of signaling pathways. The regulation of proteins of those classes was previously detected in cold-induced cysts of dinoflagellates. Guo and co-authors investigated shifts in the transcriptomic profile of *S*. *acuminata* during pellicle cyst formation in response to cold and darkness [13]. This study identified 56 DEGs related to signal transduction pathways, including MAPK, phospholipid, and calcium signaling. Notably, the differential expression of genes involved in calcium signaling was especially pronounced; specifically, a number of genes encoding calcium transport proteins were upregulated after 5 h of exposure, when the encystment was in progress. Our study revealed differential expression of calcium-binding proteins containing EF hand motif: some of them were downregulated after 7 h of cooling and other upregulated after reverting to standard cultivation conditions for 3 h. We also detected downregulation of several serine/threonine protein kinases in cold-induced cysts, and a phospholipase C (PLC)-like phosphodiesterase; PLC enzymes hydrolyze phosphatidylinositol bisphosphate into the second messengers diacylglycerol and inositol trisphosphate and are key components of various signaling cascades [30].

We revealed extensive transcriptional regulation of genes coding for pentatricopeptide repeat(s) (PPR)-containing proteins: 32 DEGs were downregulated in cold-induced cysts, while 29 DEGs were upregulated in cells after they were returned to standard cultivation conditions. In total, there were 47 differentially expressed PPR proteins, as 14 transcripts were featured in both the Cold 7 h vs. Control and Cold 7 h + ST 3 h vs. Cold 7 h comparisons.

The PPR family comprises nuclear-encoded RNA-binding proteins involved in regulating organellar gene expression [31,32]. Notably, with the exception of one differentially expressed PPR protein, all others detected in our analysis displayed homology to the provisional family, or family cluster, described as “maturation of RBCL 1”. This cluster was initially established for the MRL1 protein, a PPR protein first identified in *Chlamydomonas* and *Arabidopsis* [33]. To date, MRL1 proteins remain the only members of this provisional family. MRL1 is part of a high molecular mass complex that regulates ribulose bisphosphate carboxylase large chain (RbcL) expression. In *Chlamydomonas*, MRL1 is essential for normal accumulation of the mRNA and of the Rubisco protein, while in *Arabidopsis*, it participates either in processing of *rbcL* transcript or stabilizing the processed form [33].

We performed additional analysis of the revealed *P. cordatum* PPR proteins. Despite a certain level of similarity, the identified proteins are likely not directly homologous to MRL1. MRL1 proteins are known exclusively from the members of the green plastid lineage (Viridiplantae) and are highly conserved [33,34], while dinoflagellates belong to the red plastid lineage [35]. In different species, MRL1 contains 11–14 PPRs, comprising a PPR domain followed by the C-domain, which also contains pentatricopeptide repeats [33]. In most of the PPR proteins of *P. cordatum* that we have identified, the “PPR domain” contains only repeats ## 6 and 8–12 (as per Johnson et al. [33]), and some of the homologs also possess a part of the C-domain (Figure S2). Thus, the observed similarity may reflect a homology on a whole PPR family scale. The predicted tertiary structures of several selected proteins also correspond to the typical alternation of ɑ-helices and β-strands found in PPRs, but MRL1 appears to have a more complex folding pattern (Figure S3). Nevertheless, certain features of *P. cordatum* PPR proteins may provide insights into their possible functions. Like the MRL1 proteins, the identified homologs do not possess any functional domains other than PPRs, which corresponds to the structure of the P-class PPR proteins *sensu stricto* (as there are subclasses of the P-class) known to function in stabilizing organellar transcripts, splicing of group II introns, and activation of translation [36,37]. Thus, we assume that PPR proteins of *P. cordatum*, differentially regulated in the conducted experiments, may participate in these processes.

Other RNA-binding proteins that could be relevant to the response to cold stress, are cold shock domain proteins (CSPs). In bacteria, CSPs recover functionality of RNAs that have folded into unfavorable structures under low-temperature conditions [38]. In plants, homologous proteins have been shown to participate in both acquiring freezing tolerance and in developmental processes under normal growth conditions [38]. The identification and characterization of CSPs in *P*. *cordatum* revealed that the expression of the *PmCSP1* and *PmCSP2* genes specifically elevates in response to temperature stress, with the highest levels observed during the drop from 20 °C to 16 °C and from 20 °C to 12 °C [39]. Guo with co-authors [13] noted elevated expression of two CSP-coding genes after a 5-h incubation in cold and darkness, with no alterations observed in pellicle cysts. In cold-induced pellicle cysts of *Lingulaulax polyedra* (syn. *Lingulodinium polyedrum*), the abundance of CSPs also remained unchanged [14]. Our analysis revealed genes coding for CSP homologs in *P*. *cordatum* cysts; however, their expression did not change significantly, and they were not classified as DEGs. This finding is consistent with the aforementioned studies. Obviously, the activity of CSPs is not associated with encystment triggered by low temperatures in dinoflagellates.

MEI2 is an RNA-binding protein initially identified and studied in the fission yeast *Schizosaccharomyces pombe*, where it plays a crucial role in the transition from mitosis to meiosis [21]. Homologs of this protein have been studied in higher plants, particularly in *Arabidopsis thaliana* and *Zea mays* [22,40]. Five MEI2-like genes have been shown to be expressed in both vegetative and reproductive tissues in *A*. *thaliana* [22]. MEI2 homologs have also been revealed in various algal groups, stramenopiles, alveolates, provorans, and heteroloboseans [23,41,42].

In dinoflagellates, MEI2 homologs have been determined in the transcriptomes and genomes of various species [3,13,23,24,43,44,45,46]. Previously, we analyzed and summarized available information primarily regarding the expression of MEI2-coding genes in dinoflagellate cells, defining two putative modes of transcriptional response related to *Mei2*-like genes activity: one associated with the vegetative phase and the other with the sexual phase of the life cycle [23]. The definition of the first mode was mainly based on data concerning the differential expression of genes encoding MEI2-like proteins in *S*. *acuminata* cold- and dark-induced pellicular cysts [13] and resting cysts [24]. Expression patterns varied, revealing both upregulated and downregulated DEGs within the same dataset. Nevertheless, concomitant increases in expression of some meiosis-associated genes were observed, and the authors refrained from discussing in detail the role of MEI2-like proteins in dinoflagellate cells. Our data confirm that MEI2-like proteins do participate in the non-sexual formation of temporary cysts in dinoflagellates. Moreover, we distinguished a certain pattern of changes in the expression of detected DEGs. Among the genes suppressed in *P*. *cordatum* cold-induced cysts, some became upregulated after the transfer of cells into standard cultivation conditions, i.e., during preparation for excystment. Additionally, it was found that MEI2-like DEGs belong to two different groups of sequences. Further accumulation of data on the functional activity of MEI2-like proteins in dinoflagellate cells coupled with an assessment of their phylogenetic position can enhance the accuracy of group classification within their pool.

## 4. Material and Methods

### 4.1. Cell Culture and Experimental Design

The clonal culture of *Prorocentrum cordatum*, strain RC CCMA 0466 was maintained in artificial seawater-based f/2 medium [47] containing no silicate and sterilized by autoclaving at salinity of 17. The vitamin mixture was sterilized by sterile filtration and added separately. The batch cultures were grown under a 12 h light:12 h dark cycle (light period from 09 a.m. to 09 p.m.) at 100 μmol photons m^–2^ s^–1^ and 18 °C.

The optimal temperature and incubation time were pre-determined to achieve relatively rapid encystment of a significant proportion of the cells for subsequent sampling, as well as to obtain samples of cells preparing for excystment. The experimental culture (250 mL, cell concentration of 4 × 10^4^ cells mL^–1^) was grown for six days until the exponential growth phase was reached. Three samples were collected, with the volume calculated based on a cell count of ~2.5 × 10^6^ cells (from 38 mL to 41 mL). Two of them (samples “Cold 7 h” and “Cold 7 h + ST 3 h”) were placed in a refrigerator equipped with a lamp for algae cultivation and were additionally overlaid with ice. The temperature in the samples was 2 °C. These samples were incubated in the refrigerator for 7 h; the sample “Cold 7 h + ST 3 h” was subsequently kept under standard cultivation conditions for an additional 3 h. The third sample (“Control”) was incubated under standard cultivation conditions for 7 h. Following incubation, samples were gently mixed by rocking several times, and 1-mL of culture were taken from each sample to assess cell viability and encystment rate. The samples were then immediately centrifuged at 4500× *g* for 3 min, the supernatant was removed, and the cell pellet was rapidly frozen in liquid nitrogen and stored at −80 °C prior to RNA extraction. The experiment was performed in triplicate.

### 4.2. Light Microscopy

The encystment rate was assessed by staining the cells with Calcofluor White M2R (Thermo Fisher Scientific Remel Products, Lenexa, KS, USA), following the protocol of Matantseva with co-authors [12]. Cell viability was determined using propidium iodide staining. The proportion of cells stained with this dye, which penetrates only into dead cells [48], was counted on the preparations. Observations were conducted using a LeicaDM2500 light microscope equipped with Nomarski contrast, a fluorescent module, and a Leica DFC420 digital camera (Leica-Microsystems, Wetzlar, Germany). Light micrographs of live cells were captured at 20×, 40×, or 100× magnification. For each assessment, at least 50 cells were studied, and the percentage of stained cells was calculated.

### 4.3. RNA Extraction and Sequencing

Cells were centrifuged at 4500× g for 3 min, after which the supernatant was removed. The cell pellet was immediately frozen in liquid nitrogen and stored at −80 °C. Dinoflagellates store glucose in the form of starch [49]. To avoid starch co-precipitation with RNA, which can hinder its dissolution [50,51,52,53,54], we used a modified protocol that appears to be optimal for RNA isolation from dinoflagellate cells.

For cell lysis, we utilized a lysis buffer designed by Li and co-authors [55], which had the following composition: 1% (*w*/*v*) SDS, 400 mM NaCl, and 20 mM EDTA in 10 mM Tris/HCl (pH 8.0). Before use, the buffer was sterilized by filtering through a syringe filter, and β-mercaptoethanol was added to achieve a final concentration of 1%. The ratio of buffer volume to sample volume was 1:10 (~700 µL of buffer per sample). To disrupt thecal plates, cells resuspended in lysis buffer were subjected to beating with 1.0 mm glass beads (Sigma-Aldrich) in a FastPrep-24 homogenizer (MP Biomedicals, Irvine, CA, USA) at a speed of 5 m s^–1^ for 15 s. We then followed the extraction protocol of Li and co-authors [55]; the lysate was mixed with 0.3 volumes of saturated NaCl solution, shaken for 60 s, and centrifuged at 4 °C for 10 min. In the next step, we used silica columns to isolate and purify total RNA instead of phenol-chloroform extraction. For this purpose, we utilized the HiPure Plant RNA Kit (R415102 Magen, Guangzhou, China). Steps 1–3 of the manufacturer’s protocol, which involve cell lysis, were skipped; the upper 80% of supernatant from the previous step (centrifugation after treatment with NaCl solution) was transferred directly to a HiPure DNA column. Further RNA extraction process was conducted according to the manufacturer’s instructions. The extracted RNA was treated with DNase I (Thermo Fisher Scientific, Waltham, MA, USA). The integrity of RNA samples was assessed by 2% agarose gel electrophoresis. The purity of the samples was determined using a NanoDrop 1000 spectrophotometer (Thermo Fisher Scientific, USA).

The RNA concentration was quantified using a Qubit 4 fluorometer (Thermo Fisher Scientific, USA) and the RNA integrity was assessed using a Bioanalyzer 2100 (Agilent Technologies, Santa Clara, CA, USA). Total RNA (100 ng) from each sample was used to prepare RNA libraries. The RIN (RNA integrity number) values were >7 (average value 8.12). The sequencing libraries were prepared with TruSeq Stranded mRNA kit (Illumina, San Diego, CA, USA) following the manufacturer’s recommendations. The quality of the libraries was assessed using the Bioanalyzer system. The library preparations were sequenced using an Illumina NovaSeq 6000 platform, generating 2 × 101 bp paired-end reads. The raw sequencing data have been deposited in the NCBI Sequence Read Archive with accession numbers SAMN48699796—SAMN48699804 (BioProject ID PRJNA1267068).

### 4.4. Sequence Assembly

Demultiplexing of the sequencing reads was performed with Illumina bcl2fastq (version 2.20), adapters were trimmed with Skewer (version 0.2.2) [56]. Quality checking of the reads was conducted using FastQC v.0.11.5 [57] and the low-quality bases were clipped by Trimmomatic v.0.39 [58] with the options “SLIDINGWINDOW:4:20 MINLEN:50”. After that the remaining trimmed reads were mapped to the genomic assembly of *Prorocentrum cordatum* CCMP1329 (NCBI Assembly ID GCA_963575745.1) [25] using STAR alignment tool v.2.7.11b [59]. The locus IDs, coding sequence (CDS) IDs, and protein IDs presented in our work were provided in accordance with the assembly dataset. The read counts for the genes were obtained using htseq-count v.2.0.2 [60] with the options “-t gene -f bam -r pos -s no --secondary-alignments ignore --supplementary-alignments ignore” (Table S6).

### 4.5. Analysis of Gene Expression

The differentially expressed genes were evaluated using DESeq2 [61]. The *p*-values were corrected to control the false discovery rate (FDR). The genes with |log_2_ fold change| > 1 and the corrected *p*-values (padj) < 0.05 cutoff were assigned as differentially expressed genes (DEGs). Principal component analysis (PCA) was performed using the built-in prcomp function in R v.4.3.3 [62]. The DEGs with fold change higher than 2 were annotated using Diamond v2.1.8 and NR NCBI database with the cutoff for the maximal e-value set to 10^–5^. Besides, annotation was conducted using SMA3s v.2.1 71 with settings “-id1 30 -id2 30 -cov1 50 -cov2 50”.

OmicsBox v.3.4.5 software [63] was used for annotation of DEGs. We performed a blastp-fast search in the NCBI database with e-value < 10^−10^ cut-off and “*Polarella*” exclusion filter for taxa and additional Diamond blastp search. Then for unknown DEGs, we conducted blastp-search without exclusion filter and InterProScan search for predicted domains, sites and protein families. Additional verification of amino acid sequences was performed by search for conserved domains and motifs using the CD-Search NCBI tool against the CDD v3.21–62456 PSSMs database [64]. The DEGs were also matched by local blast to the sequences of *Polarella glacialis* reference proteome (UP000654075), which in turn were then used for functional classification in PANTHER (release 19.0) [65]. This proteome was selected as one of the three available reference proteomes for dinoflagellates in the UniProt database due to the optimality of its parameters, including protein counts, annotations, and BUSCO indicators. Gene Ontology (GO) terms describing biological processes, molecular functions, and cellular components were assigned according to *P*. *glacialis* reference proteome data.

To identify significantly enriched GO terms among DEGs, we performed GO enrichment analysis using scipy version 1.15.2, statsmodels version 0.14.4, and goatools version 1.4.12 [66] packages in Python version 3.9.0 in PyCharm 2022.2.3 (JetBrains, Amsterdam, Netherlands). All genes detected in our analysis were defined as a background set. The significance of the enrichment was assessed using Fisher’s exact test (fisher_exact function from the scipy.stats module). To control for false discovery rates, we applied the Benjamini-Hochberg correction method using the multipletests function from the statsmodels.stats.multitest module. GO terms with an adjusted *p*-value < 0.05 were considered statistically significant.

The results of the differential gene expression analysis were plotted using R v.4.3.3 [62], package ggplot2 v.3.5.2 [67]. Hierarchical cluster analysis and visualisation of its results were performed utilizing R package pheatmap v.1.0.12 [68].

### 4.6. Bioinformatic Analysis

To determine the position of revealed MEI2-like proteins, which exhibited differential expression in cold-induced cysts, among other dinoflagellate homologs, we used the set of amino acid sequences gathered from our previous work [23]. These sequences were collected from the GenBank, RefSeq, and Marine Microbial Eukaryote Transcriptome Sequencing Project [69] databases. The multiple alignment of this set and MEI2-like DEGs was carried out using MAFFT7 with the FFT-NS-i iterative refinement method (https://mafft.cbrc.jp/alignment/server/, accessed on 3 April 2025) [70]. We manually trimmed the obtained alignments in Unipro UGENE software version 52.1 [71], removing sites with more than 50% gaps and non-conserved positions. The resulting alignment was used for the selection of the evolutionary model and further phylogeny reconstruction with IQ-TREE [72] and inference with MrBayes 3.2.7a [73] in CIPRES Science Gateway [74]. In IQ-TREE, a maximal likelihood phylogenetic analysis was performed, employing ultrafast bootstrapping (10,000 replicates) and the Q.pfam+R8 evolutionary model. Bayesian analysis was conducted with 10 million generations (the stop value for the topological convergence diagnostic 0.01), LG + F + Γ_4_ evolutionary model, two runs, four Markov chains, and sampling of every 5000 chains. The phylogenetic tree was visualized in FigTree v1.4.4 (https://github.com/rambaut/figtree/releases/tag/v1.4.4, accessed on 25 November 2018). For multiple sequence alignment of MEI2-like DEGs and *Schizosaccharomyces pombe* MEI2 (CAA15822.1), we also used MAFFT7 and the FFT-NS-i method.

For a detailed analysis of PPR-containing sequences that significantly changed expression in our experiment, we retrieved the plant and green algal MRL1 amino acid sequences used in the study by Johnson et al. [33]. The multiple sequence alignment of the combined dataset was performed using MAFFT7 (FFT-NS-i method) and visualized in Unipro UGENE software. The domains and motifs were identified according to the definitions made by the aforementioned authors [33]. Additionally, we performed tertiary structure prediction of three PPR-containing proteins with different sequence features from *P*. *cordatum* and the MRL1 protein from *Chlamydomonas reinhardtii* using the AlphaFold Server (https://alphafoldserver.com/, accessed on 16 April 2025) [75].

## Data Availability

The raw sequencing data from this study have been deposited in the NCBI Sequence Read Archive with accession numbers SAMN48699796—SAMN48699804 (BioProject ID PRJNA1267068). The original datasets presented in this study are included in the article/supplementary material. Further inquiries can be directed to the corresponding author(s).

## Supplementary Materials

Following supporting information can be downloaded at: https://www.mdpi.com/article/10.3390/ijms26125432/s1.
