# Supplementary material for: Transcriptomic Analysis of Cold-Induced Temporary Cysts in Marine Dinoflagellate Prorocentrum cordatum
Source: Int J Mol Sci. 2025 Jun 6;26(12):5432. doi: 10.3390/ijms26125432 (PMC12192581; doi:10.3390/ijms26125432)
Supplement: Supplementary file 1 [file ijms-26-05432-s001.zip › Figures S1-3.pdf]

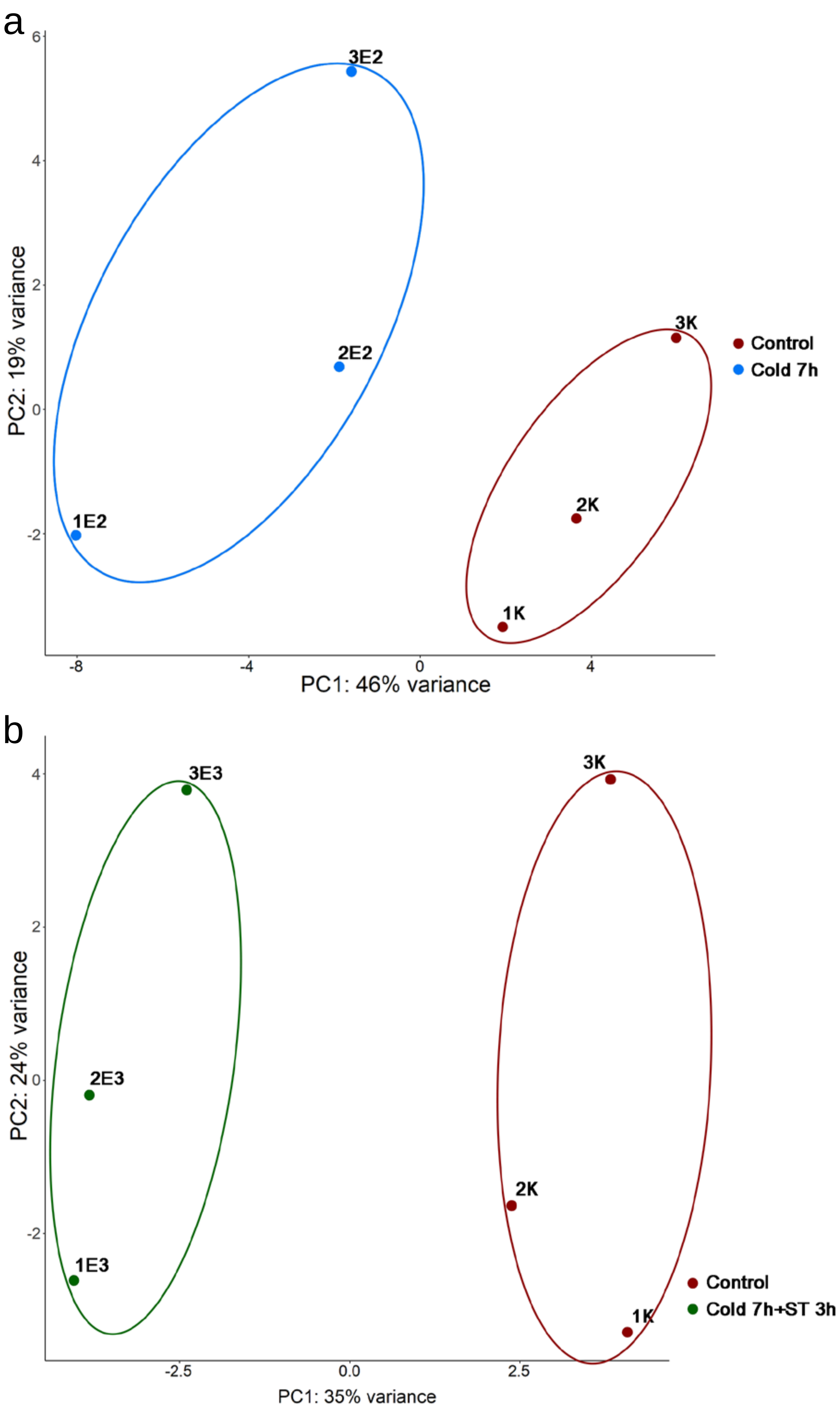

a
3E2
2E2
1E2
3K
2K
1K
Control
Cold 7h
PC2: 19% variance
PC1: 46% variance
6
4
2
0
-2
-8
-4
0
4
b
3E3
2E3
1E3
3K
2K
1K
Control
Cold 7h+ST 3h
PC2: 24% variance
PC1: 35% variance
4
2
0
-2
-2.5
0.0
2.5

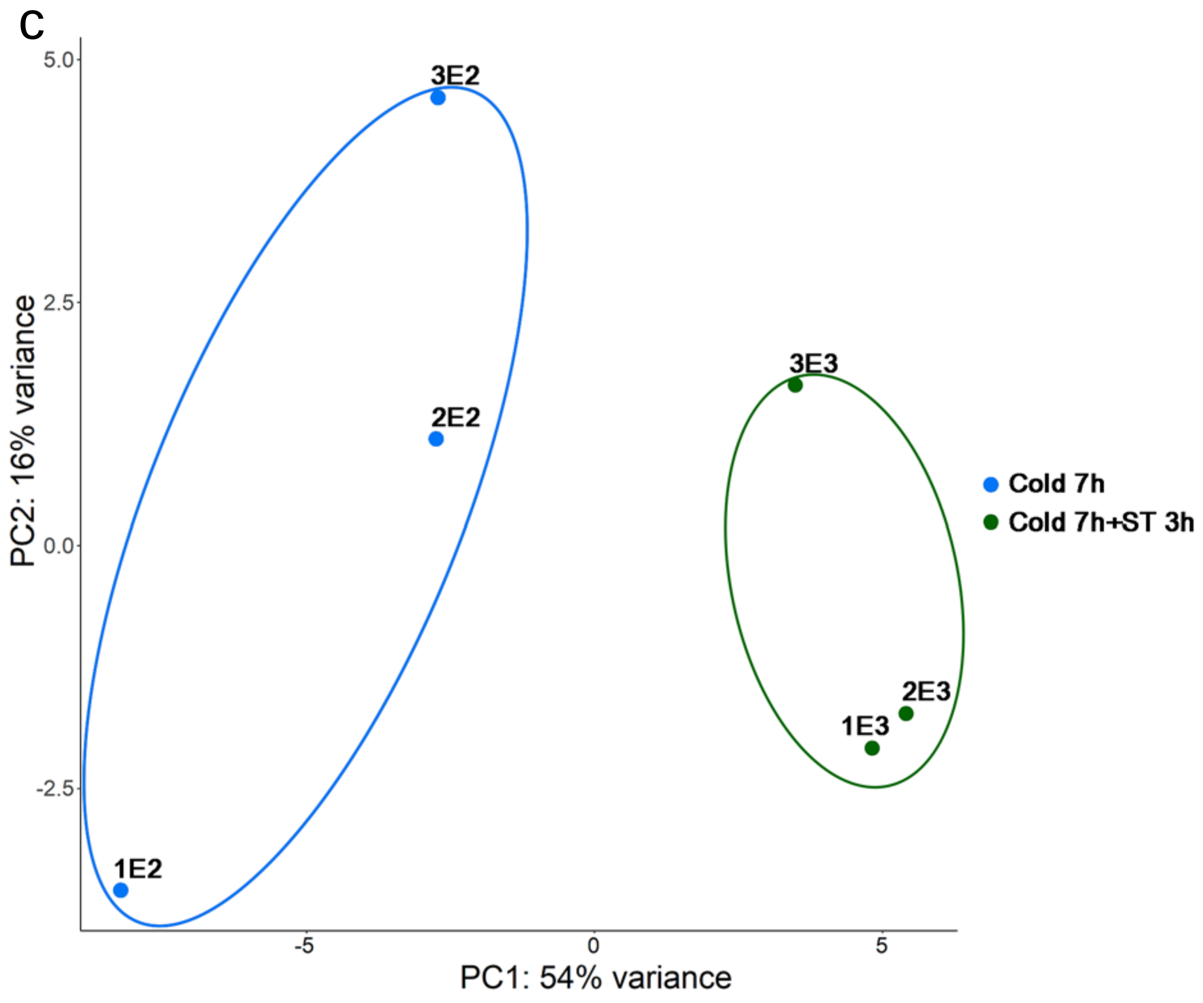

Figure S1. Principal component analysis (PCA) score plot depicting variation between the sequenced samples. **(a)** Cold 7h vs. Control, **(b)** Cold 7h+ST 3h vs. Control, **(c)** Cold 7h+ST 3h vs. Cold 7h. Control samples (n=3, “1K”, “2K”, “3K”), highlighted in red, experimental samples Cold 7h (n=3 “1E2”, “2E2”, “3E2”), highlighted in blue, and experimental samples Cold 7h+ST 3h (n=3 “1E3”, “2E3”, “3E3”), highlighted in blue, form clusters, separate from each other.

Figure S2. The fragments of multiple sequence alignment that includes *Prorocentrum cordatum* PPR-domain containing amino acid sequences, which were also determined as related to superfamily cluster PLN03218 "maturation of RBCL 1, provisional" (cl33664) by CD-search tool, and the plant and green algal sequences of MRL1 proteins retrieved from the study performed by Johnson with co-authors (2010). The *P. cordatum* sequences were encoded by genes that significantly decreased expression in comparison Cold 7h vs Control and/or increased expression in comparison Cold 7h+ST 3h vs. Cold 7h.

Plant sequences are outlined in dark green, chlorophyte sequences – in light green, dinoflagellate sequences – in blue.

The selected fragments correspond to PPR motifs (repeats) within the PPR domain and to the C-domain of MRL1 proteins. Dinoflagellate proteins exhibit similarity to PPR motifs 6, 8, 9, 10, 11, and 12 (the motifs are ranked according to Johnson et al. (2010)), as well as to part of the C-domain. The numbers 6, 8, 9, 10, 11, 12, and the caption "C-domain" at the bottom of each fragment correspond to these motifs. The boundary between motif 8 and 9 is marked with a red dashed line.

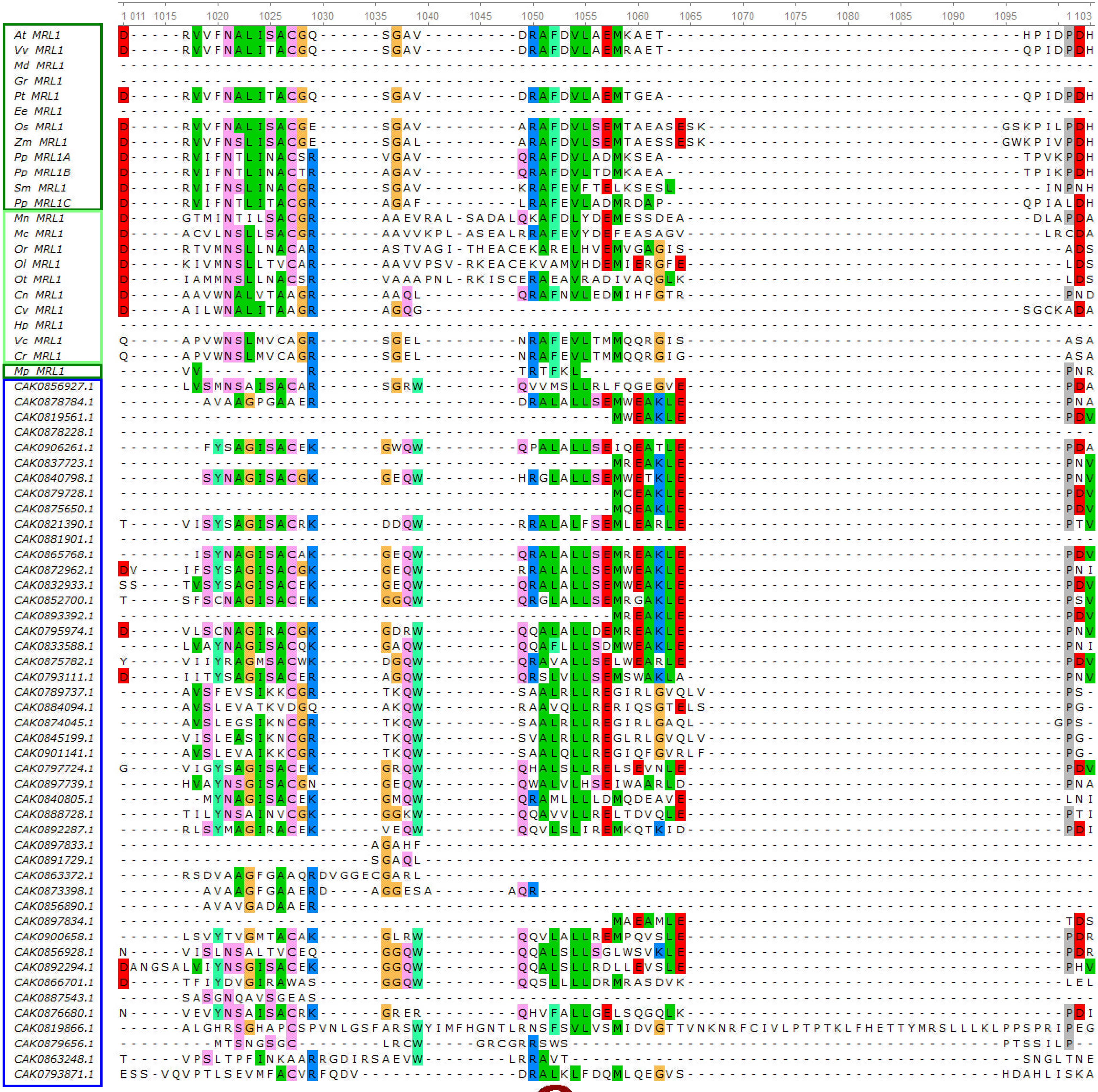
1 011
1015
1020
1025
1030
1035
1040
1045
1050
1055
1060
1065
1070
1075
1080
1085
1090
1095
1 103
At MRL1
Vv MRL1
Md MRL1
Gr MRL1
Pt MRL1
Ee MRL1
Os MRL1
Zm MRL1
Pp MRL1A
Pp MRL1B
Sm MRL1
Pp MRL1C
Mn MRL1
Mc MRL1
Or MRL1
Ol MRL1
Ot MRL1
Cn MRL1
Cv MRL1
Hp MRL1
Vc MRL1
Cr MRL1
Mp MRL1
CAK0856927.1
CAK0878784.1
CAK0819561.1
CAK0878228.1
CAK0906261.1
CAK0837723.1
CAK0840798.1
CAK0879728.1
CAK0875650.1
CAK0821390.1
CAK0881901.1
CAK0865768.1
CAK0872962.1
CAK0832933.1
CAK0852700.1
CAK0893392.1
CAK0795974.1
CAK0833588.1
CAK0875782.1
CAK0793111.1
CAK0789737.1
CAK0884094.1
CAK0874045.1
CAK0845199.1
CAK0901141.1
CAK0797724.1
CAK0897739.1
CAK0840805.1
CAK0888728.1
CAK0892287.1
CAK0897833.1
CAK0891729.1
CAK0863372.1
CAK0873398.1
CAK0856890.1
CAK0897834.1
CAK0900658.1
CAK0856928.1
CAK0892294.1
CAK0866701.1
CAK0887543.1
CAK0876680.1
CAK0819866.1
CAK0879656.1
CAK0863248.1
CAK0793871.1

6

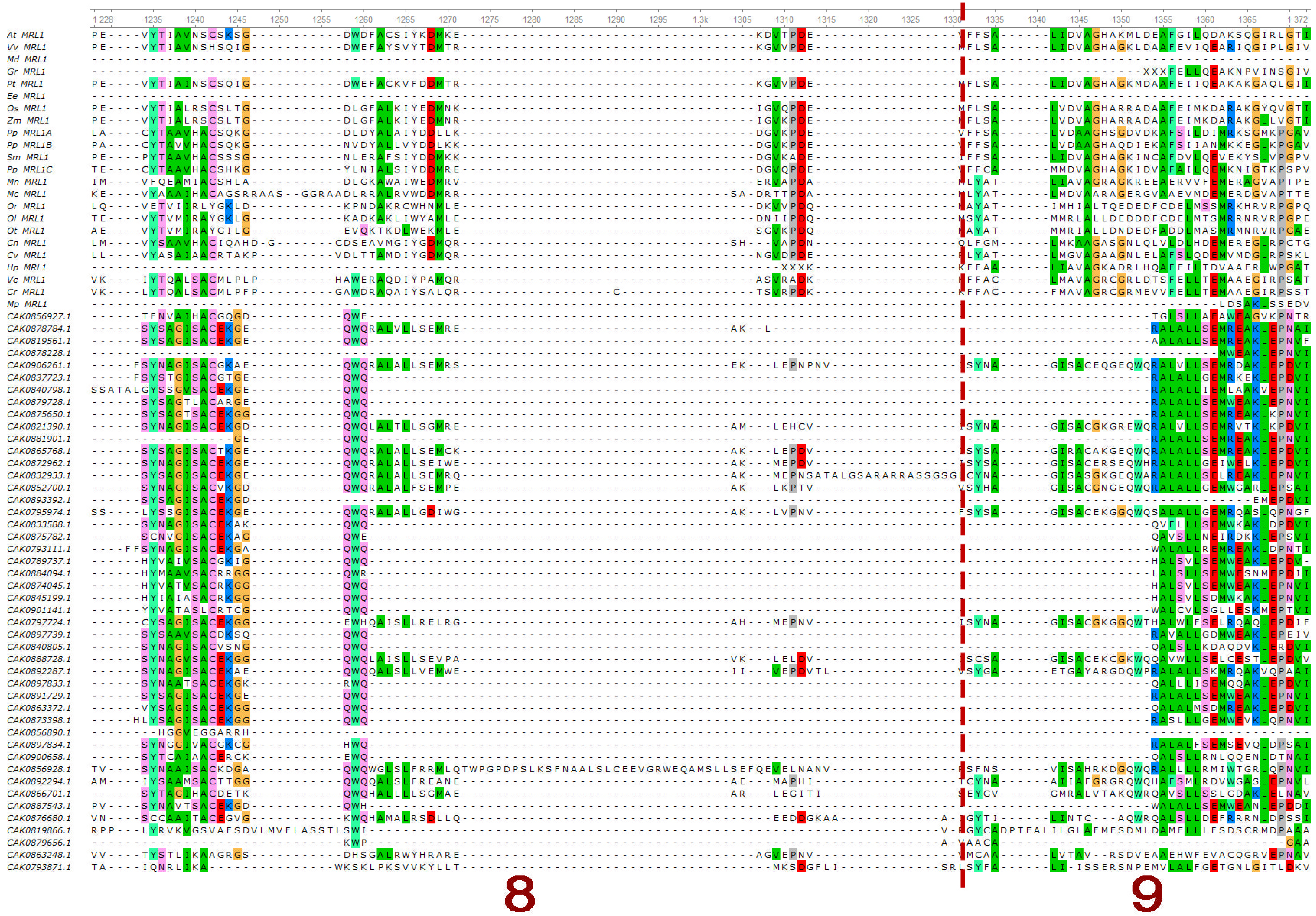
At MRL1
Vv MRL1
Md MRL1
Gr MRL1
Pt MRL1
Ee MRL1
Os MRL1
Zm MRL1
Pp MRL1A
Pp MRL1B
Sm MRL1
Pp MRL1C
Mn MRL1
Mc MRL1
Or MRL1
Ol MRL1
Ot MRL1
Cn MRL1
Cv MRL1
Hp MRL1
Vc MRL1
Cr MRL1
Mp MRL1
CAK0856927.1
CAK0878784.1
CAK0819561.1
CAK0878228.1
CAK0906261.1
CAK0837723.1
CAK0840798.1
CAK0879728.1
CAK0875650.1
CAK0821390.1
CAK0881901.1
CAK0865768.1
CAK0872962.1
CAK0832933.1
CAK0852700.1
CAK0893392.1
CAK0795974.1
CAK0833588.1
CAK0875782.1
CAK0793111.1
CAK0789737.1
CAK0884094.1
CAK0874045.1
CAK0845199.1
CAK0901141.1
CAK0797724.1
CAK0897739.1
CAK0840805.1
CAK0888728.1
CAK0892287.1
CAK0897833.1
CAK0891729.1
CAK0863372.1
CAK0873398.1
CAK0856890.1
CAK0897834.1
CAK0900658.1
CAK0856928.1
CAK0892294.1
CAK0866701.1
CAK0887543.1
CAK0876680.1
CAK0819866.1
CAK0879656.1
CAK0863248.1
CAK0793871.1
8
9

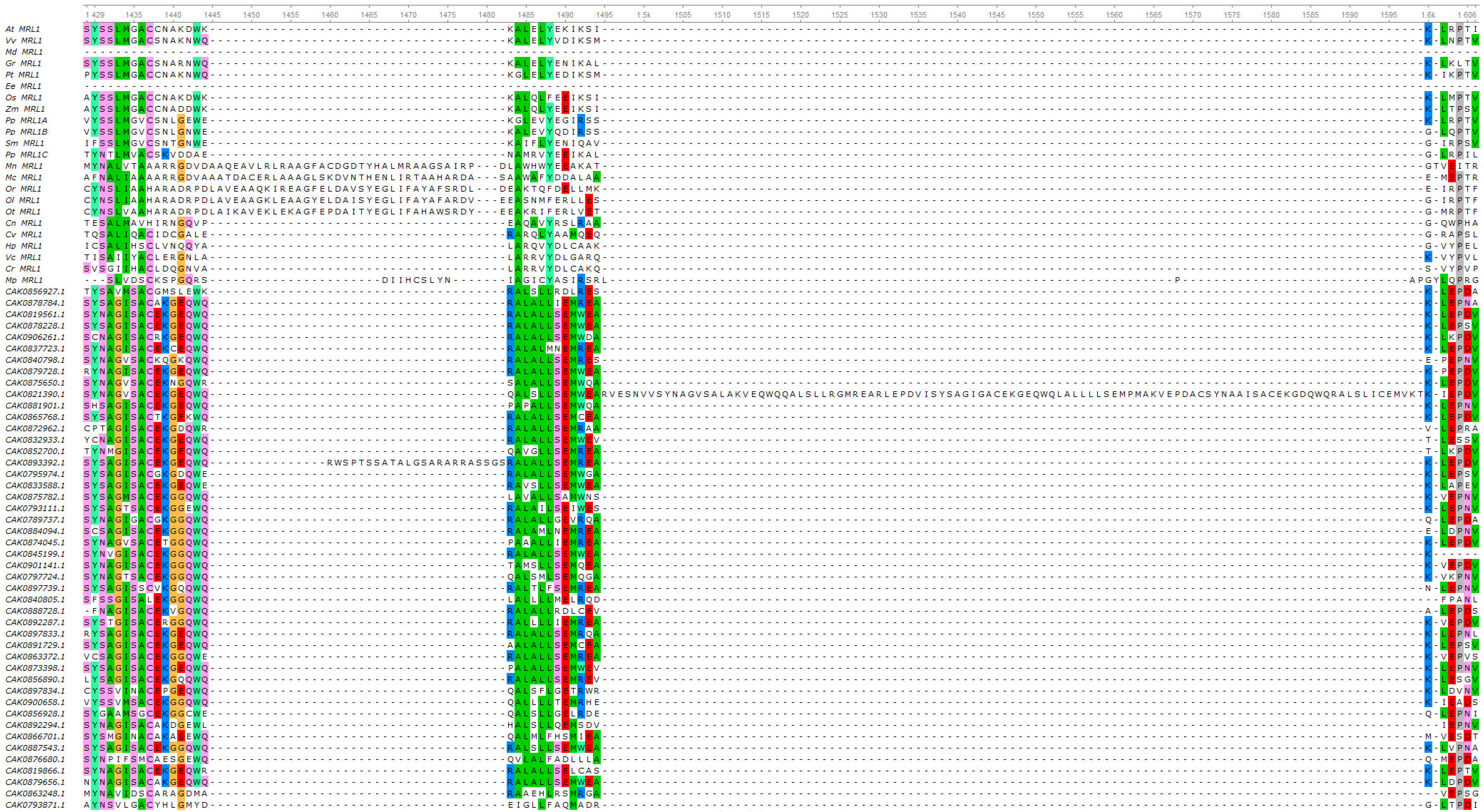
1 429
1435
1440
1445
1450
1455
1460
1465
1470
1475
1480
1485
1490
1495
1.5k
1505
1510
1515
1520
1525
1530
1535
1540
1545
1550
1555
1560
1565
1570
1575
1580
1585
1590
1595
1.6k
1 606
At MRL1
Vv MRL1
Md MRL1
Gr MRL1
Pt MRL1
Ee MRL1
Os MRL1
Zm MRL1
Pp MRL1A
Pp MRL1B
Sm MRL1
Pp MRL1C
Mn MRL1
Mc MRL1
Or MRL1
Ol MRL1
Ot MRL1
Cn MRL1
Cv MRL1
Hp MRL1
Vc MRL1
Cr MRL1
Mp MRL1
CAK0856927.1
CAK0878784.1
CAK0819561.1
CAK0878228.1
CAK0906261.1
CAK0837723.1
CAK0840798.1
CAK0879728.1
CAK0875650.1
CAK0821390.1
CAK0881901.1
CAK0865768.1
CAK0872962.1
CAK0832933.1
CAK0852700.1
CAK0893392.1
CAK0795974.1
CAK0833588.1
CAK0875782.1
CAK0793111.1
CAK0789737.1
CAK0884094.1
CAK0874045.1
CAK0845199.1
CAK0901141.1
CAK0797724.1
CAK0897739.1
CAK0840805.1
CAK0888728.1
CAK0892287.1
CAK0897833.1
CAK0891729.1
CAK0863372.1
CAK0873398.1
CAK0856890.1
CAK0897834.1
CAK0900658.1
CAK0856928.1
CAK0892294.1
CAK0866701.1
CAK0887543.1
CAK0876680.1
CAK0819866.1
CAK0879656.1
CAK0863248.1
CAK0793871.1

10

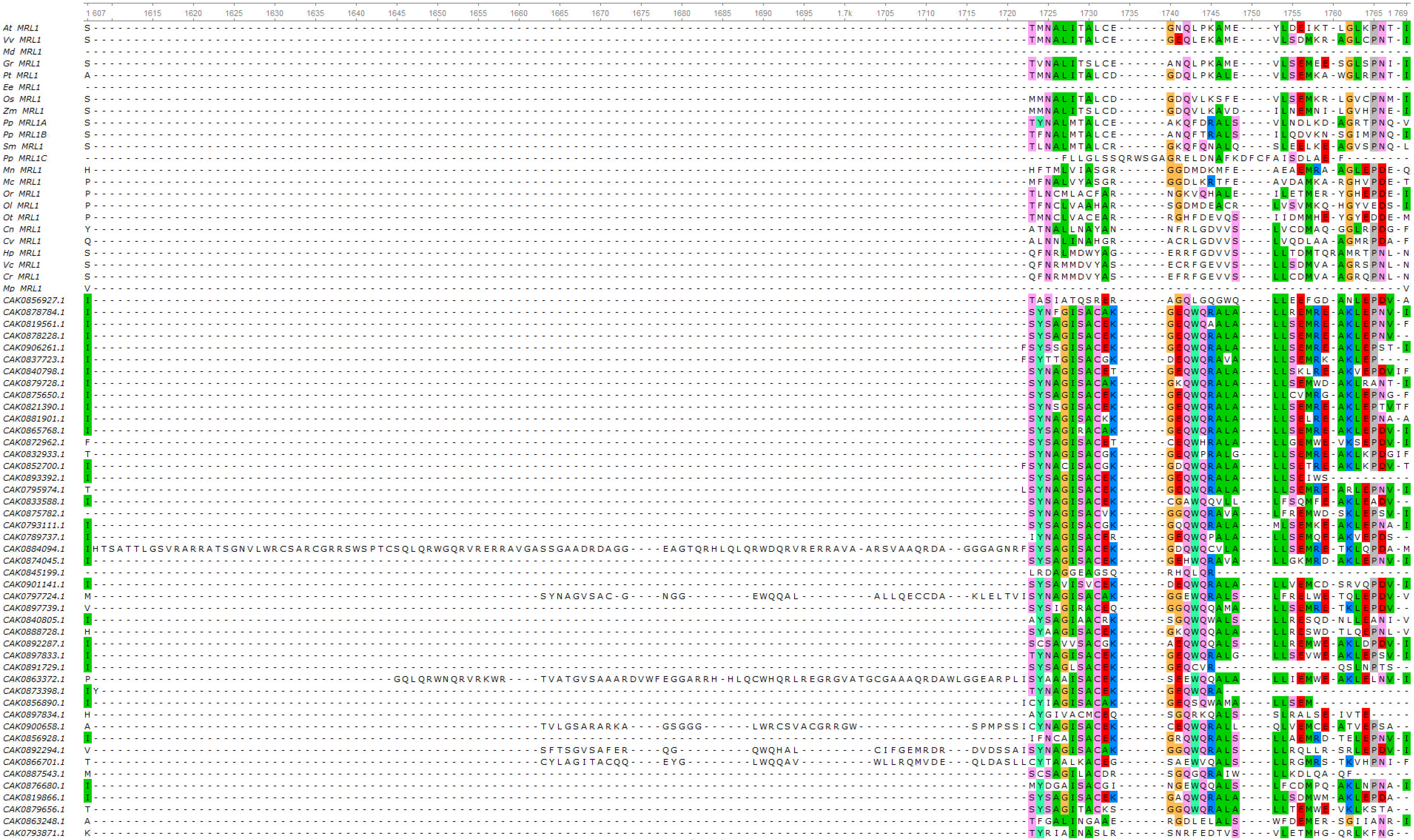

11

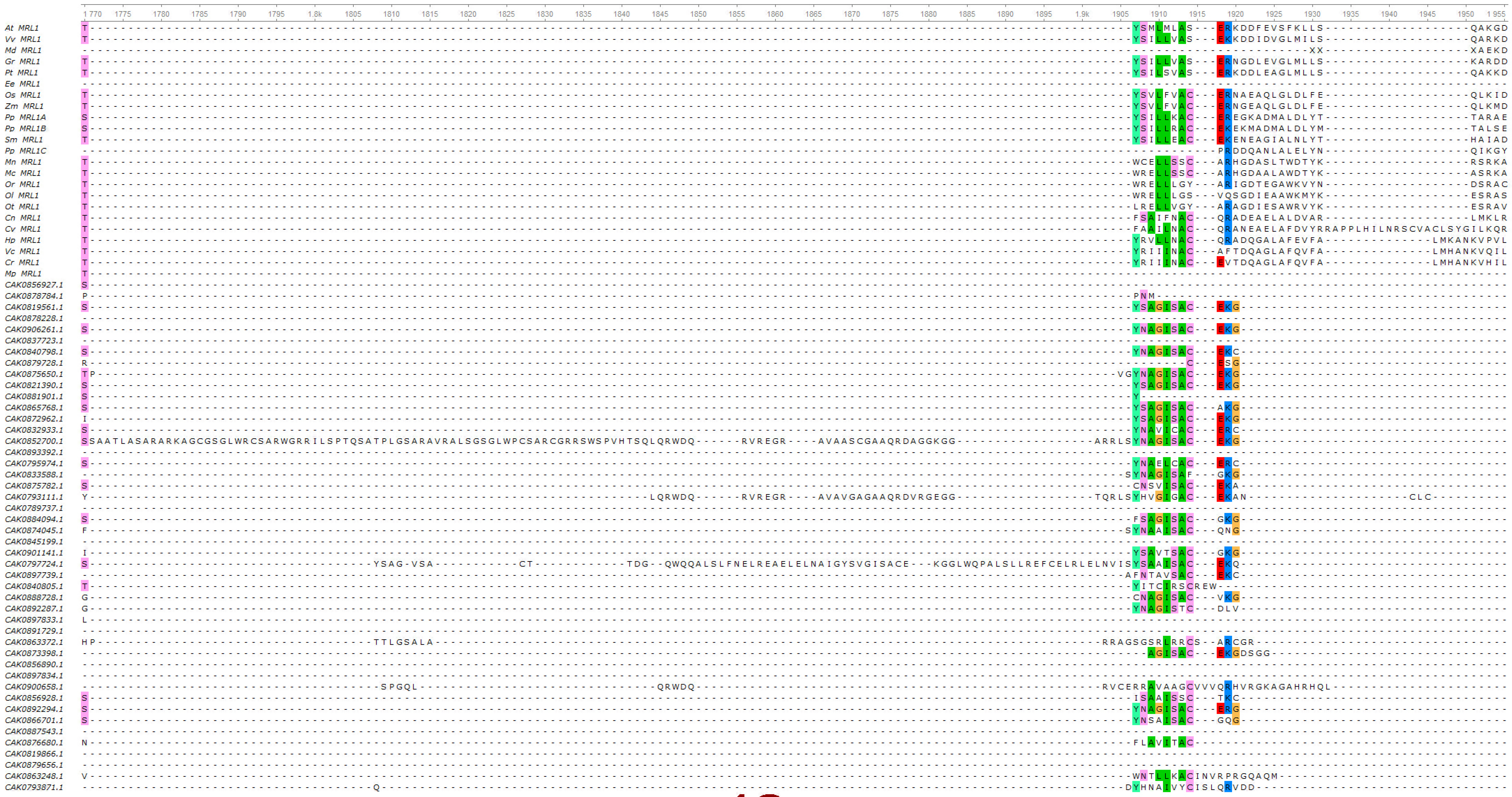

12

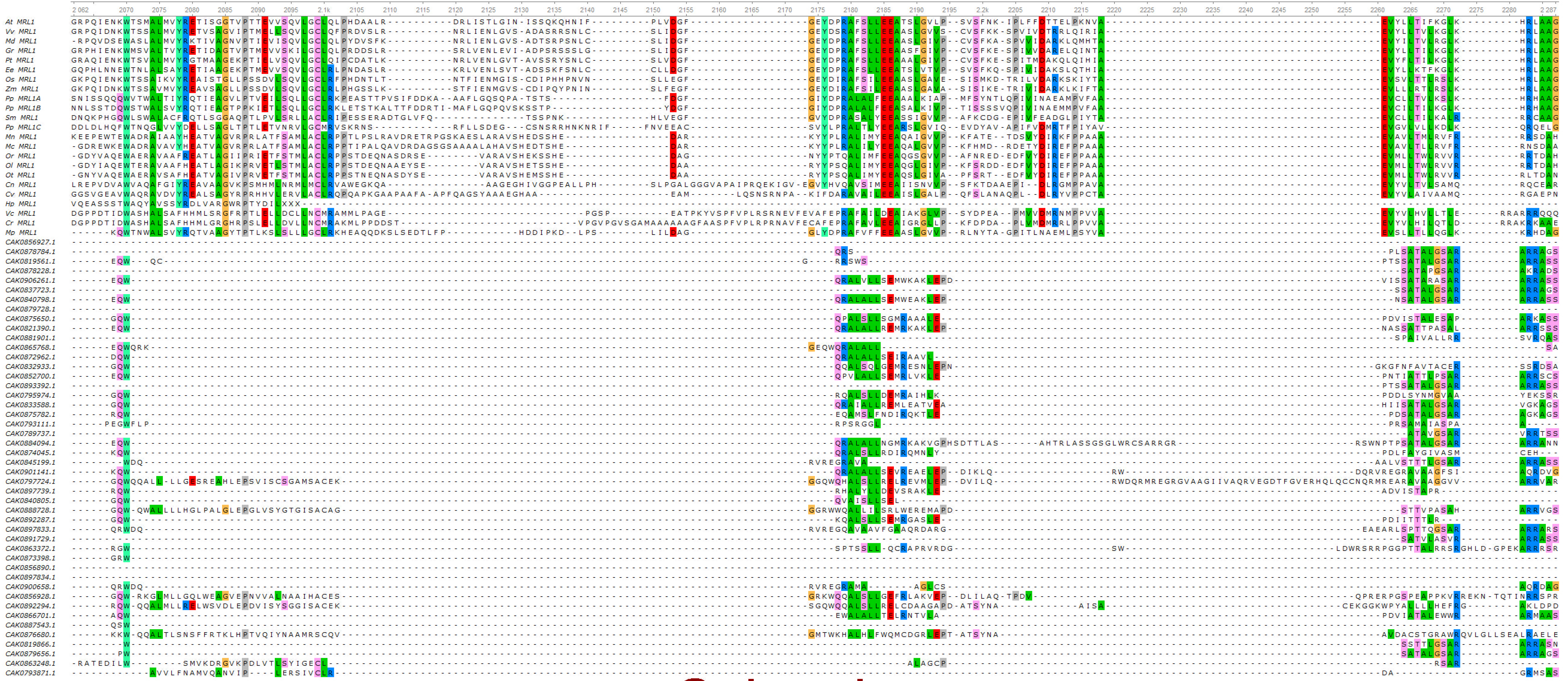
At MRL1
Vv MRL1
Md MRL1
Gr MRL1
Pt MRL1
Ee MRL1
Os MRL1
Zm MRL1
Pp MRL1A
Pp MRL1B
Sm MRL1
Pp MRL1C
Mn MRL1
Mc MRL1
Or MRL1
Ol MRL1
Ot MRL1
Cn MRL1
Cv MRL1
Hp MRL1
Vc MRL1
Cr MRL1
Mp MRL1
CAK0856927.1
CAK0878784.1
CAK0819561.1
CAK0878228.1
CAK0906261.1
CAK0837723.1
CAK0840798.1
CAK0879728.1
CAK0875650.1
CAK0821390.1
CAK0881901.1
CAK0865768.1
CAK0872962.1
CAK0832933.1
CAK0852700.1
CAK0893392.1
CAK0795974.1
CAK0833588.1
CAK0875782.1
CAK0793111.1
CAK0789737.1
CAK0884094.1
CAK0874045.1
CAK0845199.1
CAK0901141.1
CAK0797724.1
CAK0897739.1
CAK0840805.1
CAK0888728.1
CAK0892287.1
CAK0897833.1
CAK0891729.1
CAK0863372.1
CAK0873398.1
CAK0856890.1
CAK0897834.1
CAK0900658.1
CAK0856928.1
CAK0892294.1
CAK0866701.1
CAK0887543.1
CAK0876680.1
CAK0819866.1
CAK0879656.1
CAK0863248.1
CAK0793871.1

C-domain

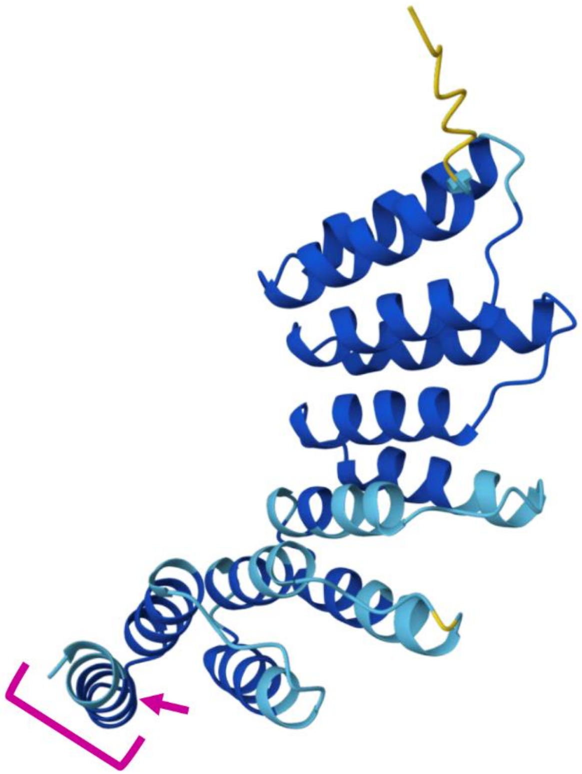

CAK0872962.1 (pTM = 0.8)

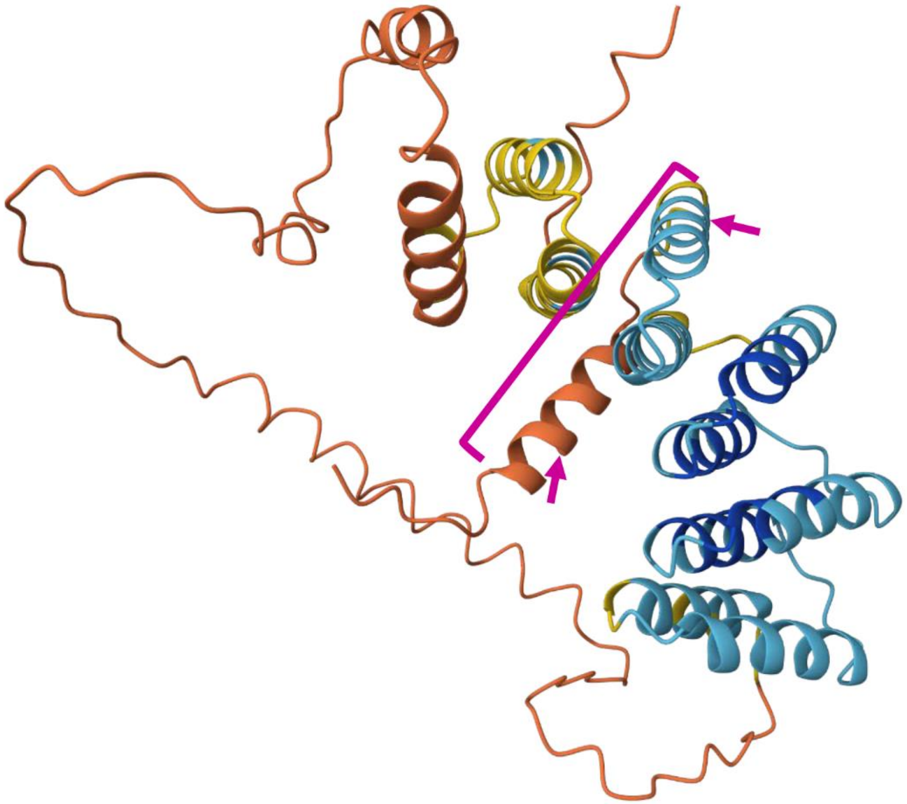

CAK0840798.1 (pTM = 0.49)

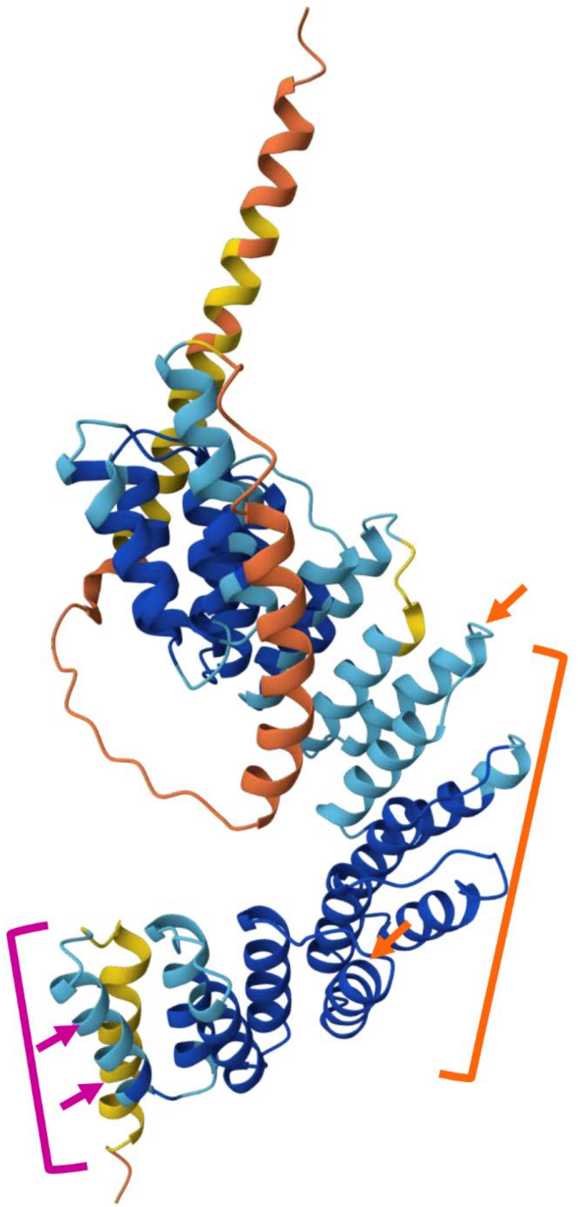

CAK0821390.1 (pTM = 0.63)

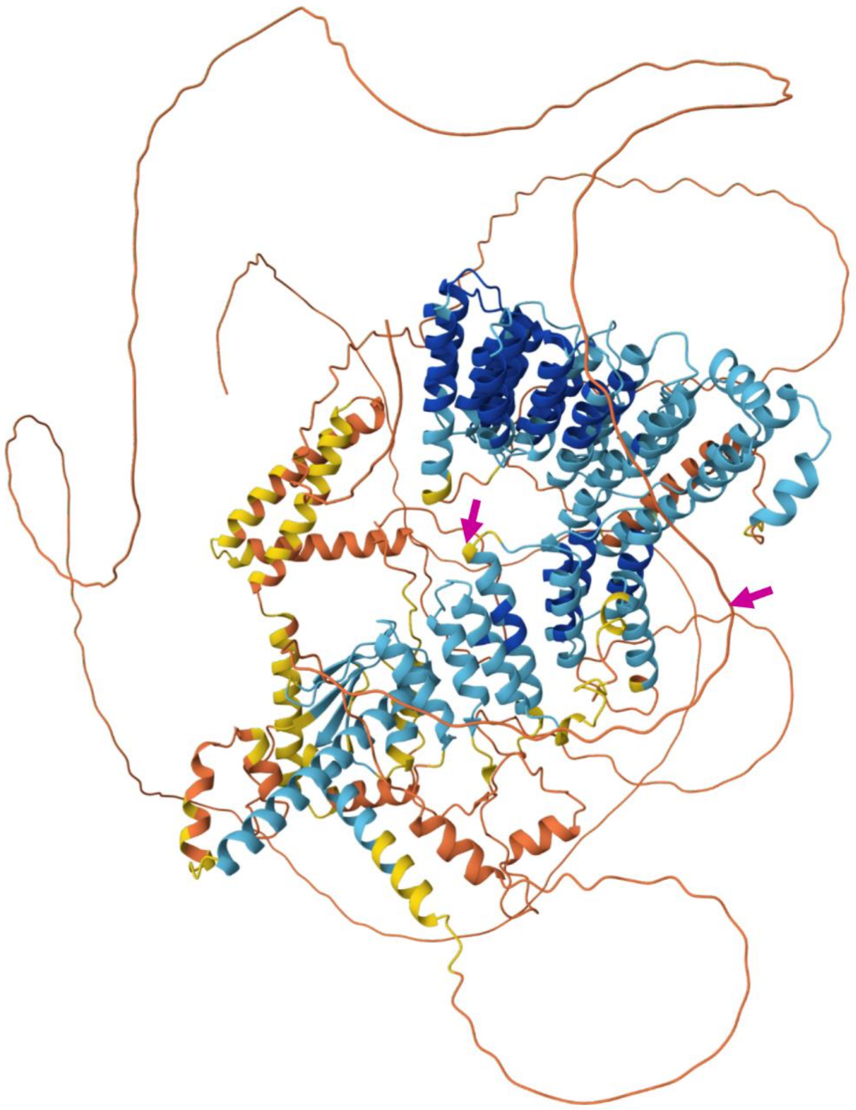

Chlamydomonas reinhardtii MRL1, sequence retrieved from Johnson et al., 2010 (pTM = 0.51)

Figure S3. Tertiary structures of three selected *Prorocentrum cordatum* homologues of MRL1 and the *Chlamydomonas reinhardtii* MRL1 protein predicted by the AlphaFold Server. The predicted template modeling (pTM) scores are indicated in parentheses. A color scheme reflecting prediction confidence is used, ranging from orange (very low, pLDDT<50) to blue (very high, pLDDT>90).

CAK0872962.1 and CAK0840798.1 sequences exhibit well-determined regions corresponding to PPR motifs 6, 8, 9, 10, 11, and 12 within the PPR domain; CAK0872962.1 has a truncated region similar to the MRL1 C-domain. CAK0821390.1 contains the aforementioned motifs but has a 105-residue insertion within PPR motif 10; the insertion is indicated by an orange bracket and orange arrows (start and end).

Regions corresponding to the MRL1 C-domain in dinoflagellate sequences are labeled with purple brackets and purple arrows (α-helices). In the C. reinhardtii MRL1 structure, the start and end of the C-domain are labeled with purple arrows.
